# Ectoparasite Community Structure of Two Bats (*Myotis lucifugus* and *M. septentrionalis*) from the Maritimes of Canada

**DOI:** 10.1155/2011/341535

**Published:** 2011-10-20

**Authors:** Zenon J. Czenze, Hugh G. Broders

**Affiliations:** Department of Biology, Saint Mary's University, 923 Robie Street, Halifax, NS, Canada B3H 3C3

## Abstract

Prevalence of bat ectoparasites on sympatric *Myotis lucifugus* and *M. septentrionalis* was quantitatively characterized in Nova Scotia and New Brunswick by making systematic collections at swarming sites. Six species of ectoparasite were recorded, including *Myodopsylla insignis*, *Spinturnix americanus*, *Cimex adjunctus*, *Macronyssu scrosbyi*, *Androlaelap scasalis*, and an unknown species of the genus *Acanthophthirius*. Male *M. lucifugus* and *M. septentrionalis* had similar prevalence of any ectoparasite (22% and 23%, resp.). Female *M. lucifugus* and *M. septentrionalis* had 2-3 times higher prevalence than did conspecific males (68% and 44%, resp.). Prevalence of infection of both genders of young of the year was not different from one another and the highest prevalence of any ectoparasite (*M. lucifugus* 64%, *M. septentrionalis* 72%) among all bat groups. Ectoparasite prevalence and intensity varied positively with roost group size and negatively with grooming efficacy and energy budgets, suggesting that these variables may be important in ectoparasite community structure.

## 1. Introduction

It is important to understand the species richness, size, and life history of ectoparasite populations to understand the biology of the host species because of the potential for ectoparasites to impact host fitness [1]. Ectoparasites are present on almost all species of mammals, including bats [2]. Most ectoparasites of bats show strong coevolutionary ties to their hosts because they never or only briefly leave the host [3–6].

Many ectoparasites are present on hosts year-round whereas others only during critical stages of the host's life cycle such as gestation or lactation. Ectoparasites may also affect host fitness, and, as expected, a strong negative correlation was found between the number of *Spinturnix psi *on the bent-wing bat (*Miniopterus schreibersii*) and the host's body condition [1]. Prevalence and intensity of ectoparasites on other small mammals and birds can vary according to several life history characteristics of the host [7, 8].

*Myotis lucifugus* and *M. septentrionalis *have several life history differences (e.g., roost group size and roost site preference), which may affect the ectoparasite community structure [9]. The little brown bat (*M. lucifugus)* regularly uses human-made structures as summer maternity roosts while the northern long-eared bat (*M. septentrionalis*) typically roosts in trees [10–14]. Little brown bats show high roost fidelity and often remain in the same roost all summer while northern long-eared bats do not show the same fidelity and often change their roost site from day to day [13, 15–17]. Males of both species roost individually or in small groups [10, 11, 18]. *Myotis lucifugus *females congregate in larger roost groups than *M. septentrionalis*, and larger number of bats coming in contact is expected to lead to a higher rate of horizontal ectoparasite transmission, thus, higher ectoparasite prevalence [3, 9, 19, 20]. Further, the relative mobility of different species of ectoparasites would facilitate the movement of more mobile species, such as bat fleas, than other less mobile species such as wing mites. Therefore, it is expected that in high bat density there will be a higher prevalence of fleas than wing mites [9, 20]. The ectoparasites of *M. lucifugus *and* M. septentrionalis *were studied in Nova Scotia, and several species of monoxenous ectoparasites were found including the wing mite (*Spinturnix americanus*), the bat flea (*Myodopsylla insignis*), and the bat bug (*Cimex adjunctus*) [21].

Mites of the genus *Spinturnix *are exclusive ectoparasites of bats within the suborder *Yangochiroptera* and complete their entire life cycle on their host [22, 23]. These mites have an extensive distribution ranging from the Nearctic to Neotropic regions [20]. *Spinturnix americanus* (wing mite) spends its entire life cycle on the patagium of the infected individual; its specially adapted claws and legs allow it to grip the wing membrane and hold onto their host, even when the bat is flying [22, 24]. 

The reproductive cycle of many species of mites is stimulated by host pregnancy hormones [1, 20]. Lactating female bats in maternity colonies host a higher intensity and prevalence of ectoparasites than nonlactating females [23, 25]. The synchronicity between host and ectoparasite reproduction allows for vertical transmission of ectoparasites to the young of the host, when they are born, who may be less efficient at decreasing ectoparasite load via grooming and may also have a naïve immunoresponse [23, 26]. Additionally, it was found that while mother-juvenile and adult-adult allogrooming occurred in their captive colony of brown long-eared bats (*Plecotus auritus*), there was no evidence of juvenile-juvenile allogrooming [27]. Once pups in the maternity colony were present, there was a shift in the existing ectoparasite population so that pups were parasitized with higher intensity until the colony dispersed [23]. Adult *M. myotis* artificially infested with *Spinturnix myotis* drastically decreased their sleep in favor of grooming [28]. This behavioral switch may be more costly for the young of the year with more restricted energy budgets [28].

*Myodopsylla insignis* (bat flea) is found from Iowa and Alberta east to Delaware and Nova Scotia [21, 29]. The bat flea is a fur-dwelling ectoparasite that feeds exclusively on the blood of its host, and its reproductive behavior is highly connected with that of its host [30]. Female rabbit fleas (*Spilopsyllus cuniculi*) are dependent on high levels of corticosteroids found in their doe rabbit host's blood to reach maturity, and these corticosteroids are only found during the last ten days of the rabbit's pregnancy and during the first five days of the newborn rabbit's life [30]. The hormones and pheromones produced by the newborn rabbits also increase sperm transfer in male fleas, and the majority of flea copulation occurs on the newborn rabbits [31]. Rothschild [32] states that this relationship could exist in bat fleas but only cites the clustering of fleas on females at the end of their winter hibernation. Although this mechanism is relatively unknown in bats, their ectoparasites are most abundant in summer maternity colonies where large groups of pregnant and lactating females congregate [1]. *Myodopsylla insignis* readily infect and are more abundant on female *M. lucifugus* than on males [20, 29]. One reason may be that, similar to rabbit fleas, bat fleas may be dependent on hormones produced by female bats to begin their own reproductive cycle [32]. Furthermore, lactating females are parasitized more often and more intensely than are their nonlactating conspecifics [20, 29]. Alternatively, or in addition, female *M. lucifugus* may host a higher intensity of fleas due to other purely mechanical reasons. Host grooming is known to be a major source of mortality for ectoparasites; nursing females have high-energy demands which may result in less time for grooming [20]. 

*Cimex adjunctus *is a large ectoparasite of bats and is closely related to the bed bug *Cimex lectularius *[24]. Bat bugs are distributed from Manitoba to Nova Scotia and south to at least South Carolina [21, 33, 34]. In the west, cimicids were found in association with the little brown bat [33]. Bat bugs are blood-feeding ectoparasites that are most abundant in the roosts of colonial bats. Adult cimicids will take bloodmeals lasting 10–15 minutes and then leave the host to digest the meal [33]. Cimicids can survive up to a year without taking a bloodmeal which allows them to stay in their host's roosts after the bats have left to hibernate [35]. 

The goal of this study was to quantitatively characterize the ectoparasite community of bats in Nova Scotia relative to host and ectoparasite life history characteristics. Specifically, we wanted to determine whether there is support for two hypotheses. First, ectoparasite prevalence and intensity vary among bat groups with different typical roost group sizes because of the variation in the potential for interhost movement of ectoparasites. Second, that ectoparasite prevalence and intensity vary between adults and young of the year because of variation in grooming efficacy. In addition to these hypotheses, we were interested in determining whether ectoparasite prevalence and intensity vary with pregnancy hormones and energy budgets of their hosts as had been suggested by others.

## 2. Methods

**Figure 1:**
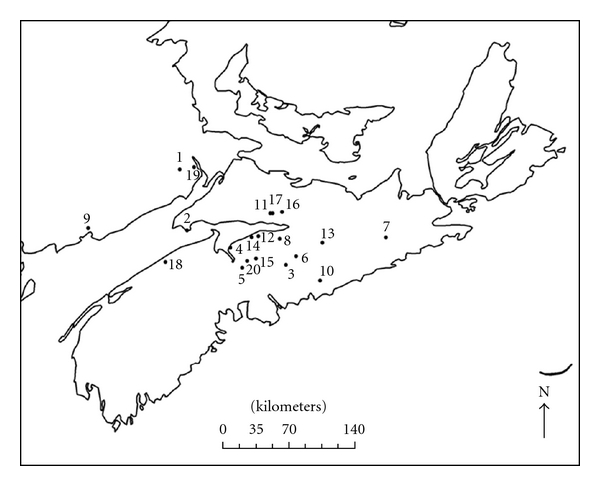
Capture sites in Nova Scotia and New Brunswick used for ectoparasite sampling from August–October, 2010. Numbers correspond with sites in Table 2.

From August 15th to October 4th 2010, bats were trapped at 17 swarming sites in Nova Scotia and three in New Brunswick (Figure 1) using harp traps (Austbat Research Equipment, Lower Plenty, VIC, Australia). During the late summer and early fall, large numbers of bats congregate around hibernacula in swarms during which they copulate and feed before ultimately entering the hibernaculum for the winter. Species, sex, and age of each individual bat were determined visually. Age was identified as either adult or young of the year based on the degree of ossification of the fourth and fifth knuckles [10, 36]. At least the first 15 bats captured each night were thoroughly and systematically surveyed in hand and checked for ectoparasites using an LED headlamp. This process involved three steps. First, the ears were examined internally at the base of the tragus and externally around the pinna. Second, the patagium was outstretched for inspection, and, due to lack of hiding places, reliable counts of ectoparasite infection were expected [24]. Finally, the fur was examined by systematically blowing on the dorsal and ventral sides of the animal. This blowing parted the animals' fur and often, if not always, disturbed ectoparasites so that they could be seen and collected. Ectoparasites were collected using a pair of stainless steel pointed tweezers and preserved in 70% ethanol. It took at least 3 minutes to check each bat for ectoparasites.

In the lab, ectoparasites were identified using keys and diagnostic characteristics [22, 24, 29]. Specifically, bat fleas *Myodopsylla insignis* (Rothschild) were identified by the presence of genal spines on the front portion of the head instead of the back, which is a trait common only to those fleas that infect bats. The maxilla is truncate, and the genal comb contains two spines. Also, the plate, which comprises the anterior portion of the head, is wide and smooth and a pronotal comb is present [24]. *Myodopsylla insignis* have a sexually dimorphic structure called the sensillum or pygidium lying laterally on the dorsum of the flea [24]. In female *M. insignis*, the sensillum is large and accessible and is located in a well-defined cuticular sensiliar plate. In the male, the sensillum is smaller and is partially covered by the anterior, overlapping segment VII [24]. *Cimex adjunctus* (Barber) (Bat Bug) have a hind femora less than 2.6 times as long as the greatest width of the femora, and bristles at the sides of the pronotum are long and thin and only slightly serrated at the tips [37]. The bristles are a diagnostic feature, as all other possible species have noticeably serrate bristles on the pronotum. *Spinturnix americanus *(Banks) were identifiable by the presence of tiny posterodorsal setae of the III and IV femora and tiny proximal dorsal setae of femora I and II [22]. Female *S. americanus* were distinguished by their idiosoma which narrows to a rounded apex posteriorly. Additionally, the presence of three or four pairs of long dorsoterminal, terminal, or ventroterminal setae was characteristic of females. Males were distinguished by their idiosoma narrowing posteriorly to a small pointed opisthosoma [22]. Ectoparasites that could not be identified in lab were sent to the Canadian National Collection of Insects, Arachnids and Nematodes for identification. 

To assess hypothesis (i), we tested the prediction that the larger roost-group sizes lead to higher rates of inter-individual ectoparasite transmission and, therefore, female *M. lucifugus *will have a higher prevalence and intensity of ectoparasites than female *M. septentrionalis*. Also, the males' solitary nature will result in a lower prevalence and intensity of any ectoparasite compared to their conspecific females. Prevalence was defined as the number individuals within a host group that were infected by at least one ectoparasite divided by the total number of individuals sampled from that host group. Intensity of infection, defined as the number of ectoparasites on one host, was calculated to characterize differences in the number of ectoparasite infecting a host. The intensity of ectoparasites on hosts throughout the population was overdispersed and not normally distributed, this is consistent with most parasite populations [38]. A one-tailed Mann-Whitney *U*-test was used to test whether the mean intensity of ectoparasites on host groups were significantly different within a 95% confidence interval [39]. Spearman's *r_s_* [38] was used to calculate the correlational value of the intensity of fleas versus. mite infection on both host species.

To assess hypothesis (ii), we tested the prediction that because of the naïve grooming behavior of young of the year [26], they will be host to higher prevalence and intensity of ectoparasites relative to conspecific adults. Prevalence and mean intensity of ectoparasites for host age groups was calculated and analyzed using a one tailed Mann-Whitney *U*-test.

Finally, to determine whether there was any pattern of association between ectoparasites with pregnancy hormones and energy budgets, we compared prevalence and intensity of ectoparasites among postlactating females and nonreproductive conspecific females captured in the same site/time period. The mean intensity of specific ectoparasites for host reproductive groups was calculated and analyzed using a one-tailed Mann-Whitney *U*-test.

## 3. Results

Fifteen or more bats were captured at 16 of the 20 capture sites, and in total 537 bats were examined for ectoparasites. In total, 599 individual ectoparasites were collected, and, of these, 597 were identified as one of five species, and two were identified to genus level (Table 1). These species included the previously recorded species for the region including *M. insignis*, *S. americanus*, and *C. adjunctus* as well as new species records for the region including *Macronyssus crosbyi *(body mite), *Androlaelaps casalis *(predatory mite), and an unknown species of genus *Acanthophthirius *(*Acanthophthirius *spp.). Of the sites where there were at least 10 bats sampled, the prevalence of bats infected by at least one ectoparasite ranged from 10% to 83% (Table 2). Of all bats sampled, 252 (47%) had at least one ectoparasite (30 adult male *M. lucifugus*, 50 adult female *M. lucifugus*, 58 young of the year *M. lucifugus*, 20 adult male *M. septentrionalis*, 21 adult female *M. septentrionalis*, 73 young of the year *M. septentrionalis*). There was no difference in ectoparasite prevalence between male and female *M. lucifugus *young of the year (64.8% and 62.9%, resp.) or *M. septentrionalis* young of the year male and females (70.7% and 72.1%, resp.), so data for both sexes were combined for all analysis.

In relation to hypothesis (i), we found the host species harbor starkly different ectoparasite communities (Table 1). Intensities of the two most abundant ectoparasites on a bat, mites and fleas, were inversely correlated with one another on both *M. septentrionalis *and *M. lucifugus *(*r*_*s*_ = −0.30, *t* = −3.6, *df* = 105, *P* = 0.0008 and *r*_*s*_ = −0.51, *t* = −6.97, *df* = 136, *P* < 0.0001, resp.). All sex/age groups of *M. lucifugus *had higher prevalence of bat fleas compared to *M. septentrionalis* counterparts. Additionally, the intensity of bat fleas (Table 3) was significantly different between host species (*Z* = 5.73, *P* < 0.0001). For wing mites this relationship was reversed with all sex/age groups of *M. septentrionalis *harboring higher prevalence (Table 1). The intensity of wing mite infection was significantly different between host species (*Z* = −4.8, *P* < 0.0001). The prevalence of any ectoparasite was virtually the same between males, but female and young of the year *M. lucifugus *had higher prevalence than *M. septentrionalis. *When examining intensities of any ectoparasite, all sex/age groups of *M. lucifugus *had higher intensities than *M. septentrionalis*, but this difference was not significantly different (*Z* = −0.03, *P* = 0.49).

Adult females consistently had higher prevalence of ectoparasites than males (Table 1). Female *M. lucifugus *were infected by any ectoparasite three times more than males; *M. septentrionalis *exhibited the same type of pattern with females being infected twice as much as males. The intensity of infection of fleas, wing mites, and any ectoparasite on male and female *M. lucifugus* was significantly different (*Z* = −3.99, *P* < 0.0001, *Z* = −2.66, *P* = 0.004, *Z* = −5.9, *P* < 0.0001, resp.). The intensities of fleas and wing mites were not statistically different between *M. septentrionalis *males and females (*Z* = −0.14, *P* = 0.44, and *Z* = −1.58, *P* = 0.057 resp.). However, when any ectoparasite intensity was compared, it was significantly different (*Z* = −2.1, *P* = 0.018).

Young of the year bats had the highest overall ectoparasite prevalence (Table 1). Specifically, of each of the *M. septentrionalis *groups, young of the year had the highest prevalence of wing mites (*S. americanus*; 62.8%), body mites (*M. crosbyi*; 7.8%), bat bugs (*C. adjunctus*; 5.9%), and predatory mites (*A. casalis*; 1%). *Myotis lucifugus* young of the year had a significantly higher prevalence of fleas (*M. insignis*; 55%) than *M. septentrionalis* young of the year. One young of the year *M. lucifugus *had 18 fleas compared to two as the maximum intensity on any young of the year *M. septentrionalis*. This relationship was reversed when examining wing mites: 62.8% of *M. septentrionalis *young of the year were infected compared to the 22% of young of the year *M. lucifugus. *The highest intensity in both host species was five. Young of the year *M. septentrionalis *had higher prevalence of any ectoparasite than either adult male, or adult female (23 and 43.8%). Young of the year *M. lucifugus *were also highly parasitized, but the difference between their adult counterparts was less pronounced; 63.7% were parasitized compared to adult male (22.1%) and adult female (68.5%). The intensity of any ectoparasite and fleas for adult *M. lucifugus *was statistically different than their young of the year counterparts (*Z* = −4.01, *P* < 0.0001, and *Z* = −3.93, *P* < 0.0001, resp.). The intensity of any ectoparasite and mites for adult *M. septentrionalis *was statistically different than their young of the year counterparts (*Z* = −6.06, *P* < 0.0001 and *Z* = −5.57, *P* < 0.0001, resp.).

Postlactating females of both species did have a higher ectoparasite prevalence (76.5%) compared to the non-reproductive females captured in the same area and time period (66%; Table 4). However, the mean intensity of infection of fleas, mites, and any ectoparasites for postlactating and nonreproductive females was not statistically different (*Z* = −0.65, *P* = 0.26, *Z* = −1.09, *P* = 0.14, and *Z* = 0.06, *P* = 0.48). The greatest difference in prevalence was seen in the bat fleas 47.1% of post lactating females were found to harbor a bat flea compared to 32% for nonreproductive females.

## 4. Discussion

The role of interspecific interactions in shaping patterns of distribution, assembly, and abundance of ectoparasite species has been broadly investigated and remains a focus of ongoing research [40]. These results suggest that the structure of ectoparasite communities and prevalence of individual ectoparasite species vary by the roost-group size, grooming efficacy of the host, and reproductive condition in bats. *Myotis septentrionalis *was host to six species of ectoparasite while *M. lucifugus *was host to only three, with *M. crosbyi *being represented by a single individual. Tello et al. [40] showed that when one species of ectoparasite has a high intensity, other species are constrained. Competitive exclusion and density compensation (negative correlations among the abundances of competing species) may be the mechanisms for this. Competition may set an upper limit to the number of individuals and ectoparasite species that can coexist. Although Tello et al. [40] examined different species of ectoparasites (*Strebla guajiro* and *Speiseria ambigua*), these species can be likened at least in habitat selection to the ectoparasites we found residing in the fur of the host (i.e., flea, body mite, predatory mite, and *Acanthophthirius spp.*). *Myotis lucifugus *hosted a higher intensity of fleas compared to *M. septentrionalis*. *Myotis septentrionalis *may be a more suitable host for the other fur-dwelling ectoparasites because they are not competing with a large numbers of fleas. The prevalence of fleas differed greatly between host and species groups. All sex/age groups of *M. lucifugus* had higher prevalence compared to their *M. septentrionalis *counterparts and both species of *Myotis *exhibited the same pattern of males being infected the least followed by females and young of the year. This is consistent with our first hypothesis that ectoparasite prevalence and intensity are affected by roost group sizes; due to *M. lucifugus' *maternity roosting behavior the fleas' reproductive strategy is facilitated and enhanced when compared to *M. septentrionalis*. The larger number of bats produce a larger amount of guano for the eggs to be laid on, and the flea's high rate of host horizontal transmission would further strengthen this difference. In *M. lucifugus* colonies, if one bat becomes too heavily parasitized and there is a threat of grooming predation, fleas may move to a less infested host. 

Prevalence of wing mites appear to be inversely correlated with the prevalence of fleas, and their intensity on hosts seem to be highly connected to the intensity of fleas on individual hosts. The difference in mite intensity between *M. lucifugus* and *M. septentrionalis *was statistically significant, and, although these ectoparasites inhabit different microhabitats on the host, their populations may still be connected. Interspecific ectoparasite load is likely a factor in this connection. Most of the bats in this study were uninfected by ectoparasites (292 of 537, 53%), and the individuals who were infected had an average intensity of infection by any ectoparasite of 2.4. Furthermore, individuals exhibiting a higher intensity of infection than the mean (77 of 237, 32.5%) were not abundant. This pattern seems logical as bats have a tolerable load, above which they become too virulent and the host must actively groom itself. 

Dick et al. [20] found that bats in close proximity had the highest rate of horizontal ectoparasite transmission. The life history of *M. septentrionalis* is one that does not facilitate horizontal transmission as much as *M. lucifugus*. Therefore, a wing mite on an individual *M. septentrionalis* is less likely to share it's host with a large number of ectoparasites. This may be a behavioral adaptation; since wing mites show much higher host affinity and do not have the same mobility as fleas, they may be easier for the host to detect. This avoidance of competition and the mite's unique physiology may allow them to reduce mortality caused by grooming. 

Males of both species were consistently less parasitized than females or young of the year. The male's solitary roosting behavior may be a behavioral adaptation to reduce ectoparasite load. A male who attempts to roost with a large number of females may have increased reproductive opportunities but will bear the added burden of a heavy ectoparasite load. Males also have a larger energy budget which they can allocate to grooming, compared to reproductive females [20]. *Myotis lucifugus* males had a lower intensity of fleas, mites, and any ectoparasites which is consistent with the hypothesis that ectoparasite prevalence and intensity will vary among groups with different typical roost group sizes. The lack of significance in *M. septentrionalis *males can be accounted for by the low intensity of ectoparasites on their conspecific females which they are being compared to. 

Young of the year of both *M. septentrionalis* and *M. lucifugus* had a higher prevalence and intensity than their conspecific adults which is consistent with the hypothesis that ectoparasite prevalence and intensity will vary between adults and young of the year. The significant difference in intensity between young of the year and adults likely stems from the differences in grooming proficiency, adult bats have an established grooming behavior, which they employ with great proficiency, and also have a stronger immunoresponse to ectoparasite bites [23]. Young of the year bats may be less aware of their ectoparasite load and less skilled at removing the ectoparasites they do detect [26]. 

Postlactating females did not have higher levels of ectoparasite intensities than their nonreproductive counterparts. However, the prevalence of any ectoparasites did differ between postlactating and non-reproductive females suggesting that ectoparasites may detect and respond to variation in pregnancy hormones and energy budgets of their hosts. The ectoparasite that showed the greatest difference between groups was the bat flea which was expected as fleas may be connected to pregnancy hormones [20, 32]. Another factor, which may, at least partially, explain this difference, is the more restricted energy budgets imposed on the females by pregnancy, lactation, and pup rearing [20]. Females must invest more energy into producing milk and caring for the pup the energy that would have been used for grooming likely gets allotted to these pup-specific energy demands [20].

## 5. New Species Records for NS

*Macronyssus crosbyi* is a monoxenous blood-feeding mite of bats [41]. These mites have a wide distribution, ranging from Texas to Eastern Canada although they have never been recorded in Nova Scotia [21, 42]. These ectoparasites can live on the host year-round as evidenced by Reisen et al. [41] who found mites actively feeding on bats during hibernation. These mites have a suppressed deutonymph stage which has become a nonfeeding, resting stage. Feeding is done exclusively by protonymphs and adults with the protonymph being a slow fixed-feeder whereas the adult is a rapid and mobile feeder [43]. 

All but one *Macronyssus crosbyi *was exclusively present on *M. septentrionalis *with no adult males being infected. Reisen et al. [41] found that *M. crosbyi *was present on significantly higher rates of females than males. It may be that, similar to fleas and wing mites, body mites infect females due to intersexual differences in amount of time spent grooming.

*Androlaelaps casalis* is a predatory mite not limited to bats; it is also often found in bird nests, grain silos, poultry houses throughout North America, Greece, and Israel [44–47]. The mite was first discovered in Italian houses in 1887 and has also been recorded on cargo ships, which may be the reason for its wide distribution [48]. Development from egg to larvae to protonymph to deutonymph to adult is considerably short (5 days) and mating occurs as soon as the adult stage is reached; however, all stages of development are predatory and actively hunt smaller mites [44]. This predatory mite feeds primarily on haemophagus and tyroglyphid mites and will also eat dried blood and eggs; if there is a lack of food, *A. casalis* will become cannibalistic and attack larvae and protonymphs [44]. In one experiment the developmental process required 27–30 prey items to be completed with adults consuming approximately 2 per day, without food adults can survive for up to 20 days before succumbing to starvation [44].

The success of *A. casalis* is likely due to its acceptance of a variety of food sources and its generalist feeding behavior [48]. If *A. casalis *requires such a large amount of prey items per day, it stands to reason that they would choose a host who is harboring enough prey for them to survive and reproduce. We found* A. casalis *exclusively on* M. septentrionalis*, a species found to harbor more acceptable prey species than *M. lucifugus*. Because of the increased ectoparasite species richness, *M. septentrionalis *may be a more attractive host for *A. casalis *than *M. lucifugus*.

Myiobiids are ectoparasites of various mammals including bats; two individual ectoparasites were found to belong to genus *Acanthophthirius* sp. (Prostigmata: Myobiidae) which is restricted to bats of the family Vespertilionidae [49]. Identifying characteristics of the genus *Acanthophthirius* in North America include expanded dorsal setae with longitudinal striations. Whitaker [42] described five of these species including *A. Eadiea condylurae*, *A. lasiurus*, *A. caudatus eptesicus*, *A. gracilis*, and* A. lucifugus*; they are characterized by an unexpanded and striated dorsal setae, setal coax IV a short spine, the ic3 (the second pair of large ventral setae, between legs 3 and 4) are closer to the body than to the middle, the ic3 thin and less than 10 *μ*m long, and the ic3 distinctly longer, respectively. However, the specimens collected in this study did not fit the description of any of these five described species from North America, and it was not possible to identify the species as the collected specimens showed a combination of characters of different species (Fred Beaulieu & King Wan Wu, Pers. Comm.), so the specimens may be a yet-to-be described species.

## Figures and Tables

**Table 1:** Prevalence of ectoparasites among the different host groups at all sample sites in Nova Scotia and New Brunswick during swarming, Aug–Oct 2010.

Ectoparasite	*Myotis lucifugus*			* Myotis septentrionalis*		
	Adult		*Young of the year	Adult		*Young of the year
	Male	Female		Male	Female	
	*n* = 136	*n* = 73	*n* = 91	*n* = 87	*n* = 48	*n* = 102
*Spinturnix americanus*	8.1%	30.1%	22%	17.2%	33.3%	62.8%
*Myodopsylla insignis*	16.2%	48%	55%	6.9%	8.3%	9.8%
*Macronyssus crosbyi*	0	1.4%	0	0	4.2%	7.8%
*Cimex adjunctus*	0	0	0	0	2.1%	5.9%
*Androlaelaps casalis*	0	0	0	0	0	1%
*Acanthophthirius spp.*	0	0	0	0	2.1%	0

*Any ectoparasite*	22.1%	68.5%	63.7%	23%	43.8%	71.6%

*There was no difference in prevalence among genders for young of the year, so they were combined for analysis.

**Table 2:** Prevalence (%) of infection (sample size) of *Myotis lucifugus* and *M. septentrionalis* with any ectoparasite at each of 20 swarming sites in Nova Scotia and New Brunswick, Aug–Oct 2010.

Site		*M. lucifugus* *(n) *	*M. septentrionalis* *(n) *	% of all bats infected
(1)	Berryton Cave (NB)	31 (29)	0 (3)	28
(2)	Cape D'Or (NS)	0 (1)	0 (4)	0
(3)	Cave of the Bats (NS)	100 (3)	67 (15)	72
(4)	Cheverie Cave (NS)	42 (26)	40 (10)	42
(5)	Frenchman's (NS)	33 (6)	45 (11)	41
(6)	Gays River (NS)	N/A (0)	10 (10)	10
(7)	Hayes Cave (NS)	74 (39)	33 (3)	71
(8)	Glenelg (NS)	44 (25)	100 (5)	53
(9)	Howes Cave (NB)	20 (5)	56 (18)	48
(10)	Lake Charlotte (NS)	83 (12)	83 (6)	83
(11)	Lear Shaft (NS)	54 (24)	25 (16)	43
(12)	Minasville Cave (NS)	33 (24)	35 (20)	34
(13)	Natural Bridge Cave (NS)	100 (2)	50 (6)	63
(14)	Peddlers Cave (NS)	N/A (0)	40 (5)	40
(15)	Rawdon Gold Mine (NS)	40 (52)	62 (60)	52
(16)	Reid Road (NS)	50 (6)	33 (3)	44
(17)	Upper Road (NS)	25 (12)	70 (10)	45
(18)	Vault Cave (NS)	15 (13)	50 (2)	20
(19)	Whites Cave (NB)	56 (9)	26 (23)	34
(20)	Woodville Cave (NS)	42 (12)	63 (8)	50

Total		46 (300)	48 (237)	47

**Table 3:** Mean intensity (standard deviation and range) of ectoparasites among the different host groups at all sample sites in Nova Scotia and New Brunswick during swarming, Aug–Oct 2010.

Ectoparasite	*Myotis lucifugus*			*Myotis septentrionalis*		
	Adult		*Young of the year	Adult		*Young of the year
	Male	Female		Male	Female	
	*n* = 136	*n* = 73	*n* = 91	*n* = 87	*n* = 48	*n* = 102
*Spinturnix americanus*	0.1 (0.6, 0–5)	0.4 (0.8, 0–2)	0.3 (0.8, 0–5)	0.3 (0.7, 0–3)	0.6 (1.2, 0–6)	1.3 (1.4, 0–6)
*Myodopsylla insignis*	0.3 (1.1, 0–10)	1.2 (1.2, 0–7)	1.7 (2.8, 0–18)	0.1 (0.3, 0-1)	0.1 (1.2, 0-1)	0.1 (0.3, 0–2)
*Macronyssus crosbyi*	0	0.01 (0.1, 0-1)	0	0	0.04 (0.2, 0-1)	0.1 (0.6, 0–5)
*Cimex adjunctus*	0	0	0	0	0.02 (0.1, 0-1)	0.1 (0.2, 0-1)
*Androlaelaps casalis*	0	0	0	0	0	0.02 (0.2, 0–2)
*Acanthophthirius spp.*	0	0	0	0	0.04 (0.3, 0–2)	0

*Any ectoparasite *	0.5 (1.3, 0–10)	1.7 (1.9, 0–7)	2.0 (2.9, 0–18)	0.3 (0.7, 0–3)	0.8 (1.3, 0–6)	1.6 (1.6, 0–6)

*There was no difference in prevalence among genders for young of the year, so they were combined for analysis.

**Table 4:** Ectoparasite prevalence, mean intensity (standard deviation and range) on postlactating females versus nonreproductive females of both species of *Myotis *in Nova Scotia and New Brunswick.

Ectoparasite	Postlactating females	Nonreproductive females
	(*n* = 17)	(*n* = 97)
*Spinturnix americanus*	41.2%, 0.5 (0.7, 0–2)	38%, 0.7 (1.2, 0–5)
*Myodopsylla insignis*	47.1%, 1.1 (1.6, 0–5)	32%, 0.7 (1.3, 0–7)
*Macronyssus crosbyi*	0	7.2%, 0.1 (0.3, 0–2)
*Cimex adjunctus*	0	0
*Androlaeps casalis*	0	1%, 0.02 (0.2, 0–2)
*Acanthophthirius spp.*	0	1%, 0.02 (0.2, 0–2)

*Any ectoparasite*	76.5%, 1.6 (1.4, 0–5)	66%, 1.5 (1.7, 0–5)

## References

[1] Lourenço SI, Palmeirim JM (2007). Can mite parasitism affect the condition of bat hosts? Implications for the social structure of colonial bats. *Journal of Zoology*.

[2] Ritzi CM, Whitaker JO (2003). Ectoparasites of small mammals from the Newport Chemical Depot, Vermillion County, Indiana. *Northeastern Naturalist*.

[3] Lučan RK (2006). Relationships between the parasitic mite *Spinturnix andegavinus* (Acari: Spinturnicidae) and its bat host, *Myotis daubentonii* (Chiroptera: Vespertilionidae): seasonal, sex- and age-related variation in infestation and possible impact of the parasite on the host condition and roosting behaviour. *Folia Parasitologica*.

[4] Christe P, Glaizot O, Evanno G (2007). Host sex and ectoparasites choice: preference for, and higher survival on female hosts. *Journal of Animal Ecology*.

[5] Dick CW (2007). High host specificity of obligate ectoparasites. *Ecological Entomology*.

[6] Bruyndonckx N, Dubey S, Ruedi M, Christe P (2009). Molecular cophylogenetic relationships between European bats and their ectoparasitic mites (Acari, Spinturnicidae). *Molecular Phylogenetics and Evolution*.

[7] Morand S, Goüy De Bellocq J, Stanko M, Miklisová D (2004). Is sex-biased ectoparasitism related to sexual size dimorphism in small mammals of Central Europe?. *Parasitology*.

[8] Fitze PS, Tschirren B, Richner H (2004). Life history and fitness consequences of ectoparasites. *Journal of Animal Ecology*.

[9] ter Hofstede HM, Fenton MB (2005). Relationships between roost prefecences, ectoparasite density, and grooming behavior of neotropical bats. *Journal of Zoology*.

[10] Davis WH, Hitchcock HB (1965). Biology and migration of the bat, *Myotis lucifugus*, in New England. *Journal of Mammalogy*.

[11] Fenton MB, Barclay RMR (1980). Myotislucifugus. *Mammalian Species*.

[12] Foster RW, Kurta A (1999). Roosting ecology of the northern bat (*Myotis septentrionalis*) and comparisons with the endangered Indiana bat (Myotis sodalis). *Journal of Mammalogy*.

[13] Broders HG, Forbes GJ (2004). Interspecific and intersexual variation in roost-site selection of northern long-eared and little brown bats in the greater fundy national park ecosystem. *Journal of Wildlife Management*.

[14] Lacki MJ, Cox DR, Dickinson MB (2009). Meta-analysis of summer roosting characteristics of two species of myotis bats. *American Midland Naturalist*.

[15] Lewis SE (1995). Roost fidelity of bats: a review. *Journal of Mammalogy*.

[16] Garroway CJ, Broders HG (2007). Nonrandom association patterns at northern long-eared bat maternity roosts. *Canadian Journal of Zoology*.

[17] Johnson JB, Edwards JW, Ford WM, Gates JE (2009). Roost tree selection by northern myotis (*Myotis septentrionalis*) maternity colonies following prescribed fire in a Central Appalachian Mountains hardwood forest. *Forest Ecology and Management*.

[18] Anthony ELP, Stack MH, Kunz TH (1981). Night roosting and the nocturnal time budget of the little brown bat, Myotis lucifugus: effects of reproductive status, prey density, and environmental conditions. *Oecologia*.

[19] Cote IM, Poulin R (1995). Parasitism and group size in social animals: a meta-analysis. *Behavioral Ecology*.

[20] Dick CW, Gannon MR, Little WE, Patrick MJ (2003). Ectoparasite Associations of Bats from Central Pennsylvania. *Journal of Medical Entomology*.

[21] Poissant JA, Broders HG (2008). Ectoparasite prevalence in Myotis lucifugus and M. septentrionalis (Chiroptera: Vespertilionidae) during fall migration at Hayes Cave, Nova Scotia. *Northeastern Naturalist*.

[22] Rudnick A (1960). A revision of the mites of the family Spinturnicidae (Acarina). *University of California Publications in Entomology*.

[23] Christe P, Arlettaz R, Vogel P (2000). Variation in intensity of a parasitic mite (Spinturnix myoti) in relation to the reproductive cycle and immunocompetence of its bat host (Myotis myotis). *Ecology Letters*.

[24] Whitaker JO (1982). *Ectoparasites of Mammals of Indiana*.

[25] Reckardt K, Kerth G (2009). Does the mode of transmission between hosts affect the host choice strategies of parasites? Implications from a field study on bat fly and wing mite infestation of Bechstein’s bats. *Oikos*.

[26] Gannon MR, Willig MR (1995). Ecology of ectoparasites from tropical bats. *Environmental Entomology*.

[27] de Fanis E, Jones G (1995). Post-natal growth, mother-infant interactions and development of vocalizations in the vespertilionid bat Plecotus auritus. *Journal of Zoology*.

[28] Giorgi MS, Arlettaz R, Christe P, Vogel P (2001). The energetic grooming costs imposed by a parasitic mite (Spinturnix myoti) upon its bat host (Myotis myotis). *Proceedings of the Royal Society B*.

[29] Smith SA, Clay ME (1988). Biological and morphological studies on the bat flea, Myodopsylla insignis (Siphonaptera: Ischnopsyllidae). *Journal of Medical Entomology*.

[30] Prasad RS (1987). Host dependency among haematophagous insects: a case study on flea-host association. *Proceedings: Animal Sciences*.

[31] Rothschild M, Ford R (1973). Factors influencing the breeding of the rabbit flea (*Spilopsylluscuniculi*): a spring-time accelerator and a kairomone in nestling rabbit urine (with notes on *Cediopsylla simplex*, another ‘hormonebound’ species). *Journal of Zoology*.

[32] Rothschild M (1965). The rabbit flea and hormones. *Endeavour*.

[33] Wilson NA, Galloway TD (2002). The occurrence of the bat bug, *Cimexpiloselluspilosellus* (Horváth) (Hemiptera: Cimicidae), in Manitoba, Canada. *Proceedings of the Entomological Society of Manitoba*.

[34] Reeves WK, Loftis AD, Gore JA, Dasch GA (2005). Molecular evidencefor novel Bartonellaspecies in *Trichobius major* (Diptera: Streblidae) and *Cimexadjunctus* (Hemiptera: Cimicidae) from two southeastern bat caves. *U. S. A. Journal of Vector Ecology*.

[35] Bartonicka T, Gailsler J (2007). Seasonal dynamics in the numbers of parasitic bugs (Heteroptera, Cimicidae): a possible cause of roost switching in bats (Chiroptera, Vespertilionidae). *Parasitology Research*.

[36] Thomas DW, Brock Fenton M, Barclay RMR (1979). Social behavior of the little brown bat, Myotis lucifugus - I. Mating behavior. *Behavioral Ecology and Sociobiology*.

[37] Usinger RL (1966). *Monograph of Cimicidae*.

[38] Roberts LS, Janovy J (2000). *Gerald D. Schmidt & Larry S. Roberts’ Foundations of Parasitology*.

[39] Sokaland RR, Rohlf FJ (1995). *Biometry: The Principles and Practice of Statistics in Biological research*.

[40] Sebastián Tello J, Stevens RD, Dick CW (2008). Patterns of species co-occurrence and density compensation: a test for interspecific competition in bat ectoparasite infracommunities. *Oikos*.

[41] Reisen WK, Kennedy ML, Reisen NT (1976). Winter ecology of ectoparasites collected from hibernating Myotis velifer (Allen) in Southwestern Oklahoma (Chiroptera: Vespertilionidae). *Journal of Parasitology*.

[42] Whitaker JO, Easterla DA (1975). Ectoparasites of bats from Big Bend National Park, Texas. *Southwestern Naturalist*.

[43] Radovsky FJ, Houck MA (1994). The evolution of parasitism and the distribution of some dermanyssoid mites (Mesostigmata) on vertebrate hosts. *Mites. Ecological and Evolutionary Analyze of Life-History Patterns*.

[44] Barker PS (1968). Bionomics of *Androlaelapscasalis* (Berlese) (Acarina: Laelapidae), a predator of mite pests of stored cereals. *Canadian Journal of Zoology*.

[45] Sinha RN, Wallace HAH (1973). Population dynamics of stored-product mites. *Oecologia*.

[46] Emmanouel NG, Buchelos CT, Dukidis CTE (1994). A survey on the mites of stored grain in Greece. *Journal of Stored Products Research*.

[47] Rosen S, Yeruhamand I, Braverman Y (2002). Dermatitis in humans associated with the mites *Pyemotestritici, Dermanyssusgallinae, Ornithonyssusbacoti* and *Androlaelapscasalis* in Israel. *Medical and Veterinary Entomology*.

[48] McKinley DJ (1963). The morphology and biology of *Haemolaelapscasalis* Berlese (Acarina; Mesotigmata). *Annals and Magazine of Natural History*.

[49] Fain A, Whitaker JO (1976). Notes on the genus *Acanthophthirius* Perkins in North America (Acarina: Myobiidae). *Bulletin et Annales de la Societe Royale Belged'Entomologie*.

